# Increased Therapeutic Efficacy of SLN Containing Etofenamate and Ibuprofen in Topical Treatment of Inflammation

**DOI:** 10.3390/pharmaceutics13030328

**Published:** 2021-03-03

**Authors:** Giuliana Mancini, Lídia M. D. Gonçalves, Joana Marto, Filomena A. Carvalho, Sandra Simões, Helena Margarida Ribeiro, António J. Almeida

**Affiliations:** 1 Faculty of Pharmacy, Research Institute for Medicines (iMed.ULisboa), Universidade de Lisboa, 1649-003 Lisbon, Portugal; giuly8210@yahoo.it (G.M.); lgoncalves@ff.ulisboa.pt (L.M.D.G.); jmmarto@ff.ulisboa.pt (J.M.); 2 LEF, Laboratórios de Estudos Farmacêuticos, 2730-269 Barcarena, Portugal; 3 Faculdade de Medicina, Instituto de Medicina Molecular ‘João Lobo Antunes’, Universidade de Lisboa, 1649-003 Lisbon, Portugal; filomenacarvalho@medicina.ulisboa.pt

**Keywords:** anti-inflammatory activity, etofenamate, ibuprofen, solid lipid nanoparticles (SLN), hydrogels, dermal delivery

## Abstract

Innovative formulations, including solid lipid nanoparticles (SLNs), have been sought to improve skin permeation of non-steroidal anti-inflammatory drugs (NSAIDs). The present study explores the use of SLNs, prepared using a fusion-emulsification method, to increase skin permeation and in vivo activity of two relevant NSAIDs: A liquid molecule (etofenamate) and a solid one (ibuprofen), formulated in a 2% hydroxypropyl methylcellulose gel through the gelation of SLN suspensions. Compritol^®^ 888 ATO and Tween^®^ 80 were used as a solid lipid and a surfactant, respectively. All production steps were up scalable, resulting in SLNs with high encapsulation efficiency (>90%), a mean particle size of <250 nm, a polydispersity index <0.2, and that were stable for 12 months. In vitro permeation, using human skin in Franz diffusion cells, showed increased permeation and similar cell viability in Df and HaCaT cell lines for SLN formulations when compared to commercial formulations of etofenamate (Reumon^®^ Gel 5%) and ibuprofen (Ozonol^®^ 5%). In vivo activity in the rat paw edema inflammation model showed that SLN hydrogels containing lower doses of etofenamate (8.3 times lower) and ibuprofen (16.6 times lower) produced similar effects compared to the commercial formulations, while decreasing edema and inflammatory cell infiltration, and causing no histological changes in the epidermis. These studies demonstrate that encapsulation in SLNs associated to a suitable hydrogel is a promising technological approach to NSAIDs dermal application.

## 1. Introduction

Non-steroidal anti-inflammatory drugs (NSAIDs) are topically applied to intact skin to treat painful inflammatory disorders, reaching effective concentrations in the tissues underlying the application site and even in the synovial fluid of joints. Topical application reduces the risk of unwanted effects by avoiding gastrointestinal (GI) disorders related to the absorption of NSAIDs in the GI tract, reducing hepatic metabolism (first-pass effect), decreasing the risk of overdosing, and increasing patient compliance [1]. Therefore, NSAIDs are widely prescribed for topical treatments in a variety of dosage forms, such as creams, gels lotions, sprays, and medicated plasters. However, these formulations have low skin permeation and may cause skin irritation that limits their extended use [2]. To overcome these drawbacks, advanced topical dosage forms have been proposed, including liposomes, transfersomes, and polymeric and lipid-based nanoparticles [3,4].

In the last decades, many authors have studied the use of solid lipid nanoparticles (SLNs) and nanostructured lipid carriers (NLCs) to increase the permeation of NSAIDs through the skin [5,6,7,8,9]. SLNs have an occlusive effect, forming a film on the skin, thus reducing water loss and increasing hydration and emollience [10]. The reduced size of SLNs increases the contact surface of drugs with the skin, thus improving NSAID permeation [4,5]. Encapsulation in lipid nanoparticles also prevents drug leakage and provides protection from the external environment [6,7].

Among the most successfully prescribed topical NSAIDs, etofenamate and ibuprofen are commercially available as creams (etofenamate), gels (etofenamate and ibuprofen), and lotions (etofenamate) (Reumon^®^ Gel, Inalgex^®^, Nurofen^®^, Ozonol^®^, and Ibuprofen gel^®^) and their inclusion in lipid nanoparticles have been extensively studied. Although ibuprofen-containing SLNs have been proposed for oral delivery for cancer chemoprevention [8], their use in dermal delivery was also studied with promising results in terms of encapsulation efficiency and sustained release properties [9,10,11,12]. Likewise, etofenamate-containing SLNs were investigated for topical application with increased in vitro anti-inflammatory effects when compared to the non-formulated drug [6,7].

In the present work, the use of SLNs was investigated as a strategy for the topical application of etofenamate and ibuprofen formulated in a 2% hydroxypropyl methylcellulose (HPMC) gel to increase skin permeation, thus combining the advantages of both systems (SLNs and gel). The novelty of this work relies on the parallel formulation of two NSAIDs, a liquid molecule (etofenamate) and a solid one (ibuprofen), through the gelation of SLN suspensions, instead of adding a preformed hydrogel. The production was an up-scalable process resulting in formulations suitable for dermal application. Physicochemical characterization and stability studies of drug-containing formulations and placebos were performed. Preclinical studies consisted of evaluating the system’s capacity to promote skin permeation of both drugs when compared to commercial medicinal products, as well as assessing in vivo the biological activity of formulations using a suitable animal model of inflammation.

## 2. Materials and Methods

Etofenamate, 2-(2-hydroxyethoxy)ethyl 2-((3-[trifluoromethyl]phenyl)amino)-benzoate was purchased from DukeChem (Olèrdola, Spain), and ibuprofen, (2RS)-2-(4-(2-methylpropyl) phenyl) propanoic acid, was purchased from BASF (Ludwigshafen, Germany). Glyceryl dibehenate (Compritol^®^ 888 ATO) was a gift from Gattefossé (Saint-Priest, France). Polysorbate 80 (Tween^®^80) was obtained from Panreac (Castellar del Vallès, Spain). Carrageenan was purchased from Sigma-Aldrich (Schnelldorf, Germany). Hydroxypropyl methylcellulose USP (HPMC) (15000 mPa.s, 2% in water at 20 °C) was obtained from FLUKA Biochemika (Buchs, Switzerland). Propylene glycol USP was purchased from Fluka (Madrid, Spain). Ethanol was purchased from Merk KgaA (Darmstadt, Germany). Menthol Ph. Eur. was obtained from Fagron (Newcastle upon Tyne, UK). Purified water was obtained with (Millipore Elix 3, Millipore SAS 67120, Molsheim, France). All other chemicals used were of analytical grade.

### 2.1. SLN Preparation

SLNs were prepared by a fusion-emulsification method as described elsewhere [6]. In short, 300 mg of lipid (Compritol^®^ 888 ATO) and different amounts of etofenamate or ibuprofen were taken in a 50 mL beaker (oil or lipid phase). The aqueous phase consisted of 10 mL of 2% (*w/w*) Tween^®^80 aqueous solution. Both the aqueous phase and the lipid phase were placed in a water bath (Water Bath VWR 6, VWR, Darmstadt, Germany) at 80–90 °C. After the fusion of the lipids, the aqueous phase was added under high stirring (Silverson SL5M, Silverson LDT Machines, Chesham, UK) at a speed of 12,500 rpm for 10 min. The preparation was removed from the bath, cooled in ice for solidification. Nanoparticles were stored at 4 °C until further use.

### 2.2. Physical Characterization of SLNs

#### 2.2.1. Particle Size and Distribution

The determination of mean particle size and polydispersity index (PdI) was made by Dynamic Light Scattering (DLS) (Zetasizer Nano S, Malvern Instruments, Malvern, UK). The samples were previously diluted 1:100 in water. Particle size was expressed as hydrodynamic diameter (nm). All measurements were made in triplicate at room temperature.

#### 2.2.2. Zeta Potential

The determination of the surface electrical charge (expressed as zeta potential) was performed by measuring the electrophoretic mobility of SLNs (Malvern Instruments 2000, Malvern, UK). Samples were diluted 1:100 in water. All measurements were made in triplicate at room temperature.

### 2.3. Thermal Analysis

#### 2.3.1. Differential Scanning Calorimetry (DSC)

Calorimetric analysis was performed on a differential scanning calorimetry (DSC) Q200 calorimeter (TA Instruments, New Castle, USA). About 3 mg of the SLN dispersions and their constituents (Compritol^®^ 888 ATO and Tween^®^80) were rigorously weighed in an aluminum pan and subsequently hermetically sealed. Measurements were made against the empty reference pan at heating intervals of 10 to 125 and 125 to 10 °C, with a heating/cooling speed of 5 °C/min.

#### 2.3.2. Dynamic Light Scattering (DLS)

Thermal analysis by dynamic light scattering (DLS) was performed using Zetasizer Nano S apparatus (Malvern Instrument, Malvern, UK), equipped with a Peltier precision temperature control unit (with a 0.1 °C accuracy). A He-Ne laser with λ = 633 nm was used as the light source. The study of the influence of the temperature variation on the mean particle diameter was determined between 25 and 90 °C (heating phase) and between 90 and 25 °C (cooling phase), with a velocity of heating/cooling of 0.5 °C/min evaluating the mean particle diameter, the PdI, and the total dispersion intensity. Samples were previously diluted 1:100 in water.

### 2.4. Transmission Electron Microscopy (TEM)

Morphology analysis was carried out according to a previously described TEM method, using a Hitachi H-8100 (Japan) microscope equipped with a microanalysis system by X-ray energy dispersion spectrometry and with an element detector ThermoNoran (Madison, WI, USA) [13]. An aliquot of the nanoparticle suspension was applied to a copper grid and dried at room temperature.

### 2.5. Atomic Force Microscopy (AFM)

The analysis of SLNs by atomic force microscopy (AFM) was performed using an Axiovert 200 inverted microscope (Carl Zeiss, Jena, Germany) containing a NanoWizard II system (JPK Instruments, Berlin, Germany). The system is equipped with a piezoelectric scanner with a linear z-range of 15 µm and an infrared laser. SLN formulations were diluted (1:100) in purified water and placed over a glass slide treated with poly-L-lysine. After 20 min, the preparation was washed with water and dried at room temperature. The analysis of the SLN was performed using the intermittent tapping method, in which the probe tip touches the surface of the sample to be analyzed discontinuously. Data were obtained using sharp silicon oxide tips with a 6 nm tip radius, a resonant frequency of about 60 kHz, and a constant energy of 3 Nm-1. Data were analyzed with the JPK v3 processor (JPK Instruments, Berlin, Germany).

### 2.6. Encapsulation Efficiency

The encapsulation efficiency (EE%) of etofenamate and ibuprofen was determined directly by measuring the drug concentration in the nanoparticles. To separate non-incorporated drug, PD-10 exclusion chromatography columns (Bio-Rad Laboratories, Hercules, USA) were used. In summary, 2.5 mL drug-loaded SLN suspension was applied to the top of the column and eluted with 3.5 mL of water. To 500 μL of the eluted fraction 500 μL of 1% (*v/v*) aqueous solution of octyl phenoxy polyethoxyethanol (Triton X100; AppliChem, Darmstadt, Germany) and 8% sodium lauryl sulfate (SDS; Sigma-Aldrich, Madrid, Spain) were added. The resulting mixture was heated at 60 °C for 2 min, cooled, and then diluted 1:10 with methanol. After vigorous vortexing for 5 min, the mixture was centrifuged at 4000 rpm, for 10 min, at 4 °C, and the lipid sediment was rejected. Incorporated drug was calculated quantifying the drug in the supernatant by high-performance liquid chromatography (HPLC) using the methodology described in Section 2.7. The EE% was then calculated according to the following equation:

EE% = [encapsulated drug]/[total drug] ×100(1)

### 2.7. Etofenamate and Ibuprofen Quantification

Quantification of etofenamate and ibuprofen was performed by HPLC using the following chromatographic conditions: Waters Nova-Pack C18 Column, 250x4.6 mm, 4 μm; the mobile phases consisted, for etofenamate, of a mixture of methanol:acetonitrile:water (45:35:20) containing 1% concentrated phosphoric acid (pH 6.0), and, for ibuprofen, of a mixture of methanol:acetonitrile:water (45:30:25) containing 1% concentrated phosphoric acid (pH 6.5); the flow rate used was 1 mL/min, with a running time of 10 min, and the injection volume was 20 μL. Detection by UV absorption was at 285 nm for etofenamate and 200 nm for ibuprofen.

### 2.8. In Vitro Cytotoxicity and Internalization Studies

Cytotoxicity was evaluated in vitro using fibroblast and keratinocyte cell lines, in which cell viability was assessed by reduction in MTT (3-(4,5-dimethylthiazol-2-yl)-2,5-diphenyltetrazolium bromide) [3,14,15]. Human dermal fibroblast cell line (Df; ZenBio, Inc., Research Triangle, NC, USA) and human keratinocytes (HaCaT; CLS, Hamburg, Germany) were cultured in 96-well plates with 100 µL/well of RPMI-1640^®^ medium (Gibco, Loughborough, UK) supplemented with 10% fetal bovine serum (FCS, Life Technologies, Inc., Loughborough, UK), penicillin (100 IU/mL) and streptomycin (100 μg/mL), with a cell density of 2.5 × 10^4^ cells per well (wet atmosphere 95% and 5% CO_2_, at 37 °C). After 24 h of incubation 10 µL of each sample, i.e., empty SLNs (designated as SLN), ibuprofen loaded-SLNs (SLN Ibu; 3 mg/mL), etofenamate loaded-SLNs (SLNs Eto; 6 mg/mL), etofenamate in DMSO (Eto; 1.2 mg/mL), ibuprofen in DMSO (Ibu; 3 mg/mL), and DMSO, were added to each well, with 9 replicates. The culture medium was used as a negative control. As a positive control, an SDS solution (1 mg/mL) was used. After 24 h of incubation, the culture medium was replaced with fresh media containing 0.5 mg/mL of MTT. After 3 h of incubation, the medium was carefully removed, and the intracellular formazan crystals were solubilized and extracted with 100 µL/well of dimethyl sulfoxide (DMSO). After incubation, absorbance at 570 nm was measured in a plate reader (FLUOstar Omega, BMG LABTECH, Germany). Untreated cells were used as a control with 100% viability. The relative cell viability (%) was calculated using the following equation:

Cell viability (% of control) = ([Absorbance] sample)/([Absorbance] control) × 100(2)

Cell internalization was assessed according to the methodology described elsewhere [16]. SLNs were marked with Nile red (Sigma-Aldrich, Spain). The HaCaT cell line was grown on a 4-chamber glass slide (Nunc ™ Lab-Tek ™ II Chamber slide ™ system, Scientific Term, Loughborough, UK), in RPMI-1640^®^ medium (Gibco, Loughborough, UK) supplemented with 10% serum fetal bovine (FCS, Life Technologies, Inc., Loughborough, UK), penicillin (100 IU/mL) and streptomycin (100 μg/mL). Cells were then exposed to the formulations for 1 h, i.e., empty SLNs, SLNs containing ibuprofen, and SLNs containing etofemanate, at the same concentrations used in the cytotoxicity assay. After incubation, cells were washed with 10 mM isotonic phosphate buffer (PBS) containing 10 mM glycine, fixed for 15 min, protected from light, with a 4% (*m/v*) solution of paraformaldehyde, and were washed three times with PBS. Slides were mounted with a coverslip containing mounting medium (Prolong Antifade, Invitrogen, Loughborough, UK), with 4′,6–diamidino-2-phenyl-indole (DAPI), for the labeling of nuclear DNA. Cells were then observed and photographed under a Zeiss Axioskop 4.0 fluorescence microscope (Zeiss, Oberkochen, Germany).

### 2.9. Preparation of Hydrogels

Hydrogels with and without SLNs were prepared according to the compositions shown in Table 1 at room temperature. SLNs and drug-loaded SLNs were prepared by dispersing the aqueous thickening agent (HPMC), propylene glycol, ethyl alcohol, and menthol into previously prepared SLN suspensions under magnetic stirring (400 rpm for 12 h) until gel formation. The gels were packed in aluminum tubes.

### 2.10. Characterization of the Hydrogels

The macroscopic appearance of each formulation was evaluated through the organoleptic characteristics and used as the first indicator of stability.

Apparent viscosity and rheological profile were evaluated using a Brookfield Rotation Viscosimeter (RV DV-II, SSA, spindle SC27 (Brookfield Engineering Laboratories, Inc., Middleborough, MA), at room temperature. The shear rate (1/s) versus shear stress (Pa) plots were obtained by submitting the samples to a shear rate sweep from 0.6 s^−1^ to 24.47 s^−1^ and backwards during 3.75 min.

The pH of each formulation was assessed with the pH meter (pH 744, Metrohm^®^, EUA) at room temperature. Three measurement replicates were performed.

Particle size analyses of SLNs incorporated in hydrogels and drug quantification were performed as described in Section 2.2.1 and Section 2.7, respectively.

In vitro cytotoxic potential of drug-loaded SLN hydrogels was evaluated as described in Section 2.8.

Hydrogels were assessed for antimicrobial preservation efficacy and microbiological examination of non-sterile products (Ph. Eur. 5.1.3 and 2.6.13, respectively [17]).

### 2.11. Stability Studies

The stability of SLN-containing hydrogels was evaluated according to the ICH Q1A (R2) guideline [18]. Real-time studies at 25 ± 2 °C/60 ± 5% relative humidity (RH) for 12 months, and accelerated studies at 40 ± 2 °C/75 ± 5% RH for 6 months, were performed using three batches. The sampling plan followed the recommendations of the guideline, and the parameters evaluated were organoleptic characteristics, particle size, pH, rheological properties, and microbiological control.

### 2.12. In Vitro Studies of Percutaneous Absorption

To evaluate the release and permeation of etofenamate and ibuprofen from SLNs and SLN-containing hydrogels, Franz diffusion cells were employed. Two reference drug products were used for comparison: Reumon^®^ Gel 5% (Bial-Portela & Cª., SA, Trofa, Portugal; etofenamate reference gel) and Ozonol^®^ 5% (Omega Pharma Portuguesa, Portugal, Portugal; ibuprofen reference gel).

#### 2.12.1. Release Studies

Drug release from SLN and SLN-containing hydrogels was measured using hydrophilic polysulfone membranes with 0.45 µm pore (Tuffryn^®^, Pall Corporation, New York, NY, USA). The membranes were washed and equilibrated with ethanol/phosphate buffer pH 7.4 (40:60 *v*/*v*) for 30 min. Then, they were mounted between the donor and receptor compartments in static vertical Franz cells (3 mL receptor solution volume; diffusion area of 1 cm^2^).

Ethanol/phosphate buffer pH 7.4 (40:60 *v/v*) was used as the receptor phase that assured perfect sink conditions during all experiment periods and, it was continuously stirred at 200 rpm with a small magnetic bar, and the temperature was maintained at 32 ± 0.5 °C. Samples (200 μL) of each formulation were uniformly applied on the membrane surface facing the donor compartments, which were immediately covered with Parafilm^®^ to avoid evaporation. Samples (200 μL) were withdrawn periodically at 1, 2, 3, 4, 5, and 6 h from the receptor compartments of the Franz cells and replaced by the same volume of receptor medium. At least 6 cells were used for each formulation, and the drug concentrations in the collected samples were quantified by HPLC. Release kinetics was evaluated according to the following mathematical models [19]:

(1) Zero-order model: Q_1_= Q_0_ + K_0_t, where Q_1_ is the amount of drug dissolved in time t, Q_0_ is the initial amount of drug in solution, and K_0_ é is the zero-order release constant;
(2) Higuchi model: Q_t_= K √t, where Q_t_ is the amount of drug released in time t, and K is the Higuchi release constant.

#### 2.12.2. Permeation Studies

Drug permeations were determined in infinite dose conditions, using human skin obtained surgically from the abdominal region of a healthy 63-year old female Caucasian from a esthetic clinic. This protocol respected the Helsinki Declaration and Good Clinical Practice studies on topical products. The skin sample was treated in isotonic phosphate buffer, pH 7.4 at 60 °C for 30 sec, to separate the stratum corneum (SC). This was visually inspected to detect possible defects and was cut in sections of adequate dimension for Franz cells. Assay conditions were the same as described in the previous section. Samples were collected immediately after the application (t = 0) and periodically at 1, 2, 4, 6, 8, and 24 h. Permeability coefficients (Kp, cm h^−1^) were calculated by dividing the permeation flux (in μg cm^−2^ h^−1^) by the initial drug concentration in the donor compartment (in μg cm^−3^), by applying the Fick’s second law of diffusion and assuming that in sink conditions the concentration of drug in the receptor compartment is negligible in comparison with that of the donor compartment.

### 2.13. Anti-Inflammatory Effect of Ibuprofen-SLN and Etofenamate-SLN Gels

All animal experiments were conducted in line with the recommendations of the animal welfare board (ORBEA) of the Faculty of Pharmacy, Universidade de Lisboa, as well as approved by the competent national authority Direção-Geral de Alimentação e Veterinária (DGAV) (Protocol EXPL/DTP-FTO/0308), and in accordance with the EU Directive (2010/63/EU), the Portuguese laws (DL 113/2013, 2880/2015, 260/2016 and 1/2019), and all relevant legislation.

**Figure 1:**
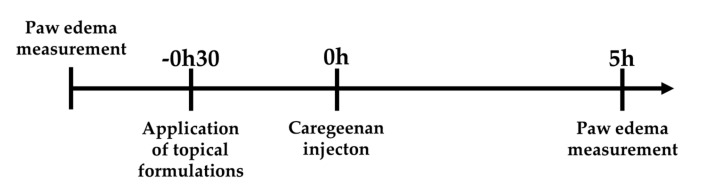
Carrageenan edema model time course.

To assess the anti-inflammatory activity of the developed formulations, we used the carrageenan paw edema model in rats (Figure 1) [20] as it is one of the most used tools to study acute local inflammation by measuring the percentage of edema after carrageenan paw injection. Animals were acclimatized before the experiments and housed in plastic cages under standard laboratory conditions, fed commercial chow, and acidified drinking water ad libitum. Male Wistar rats of 8 weeks old (Charles River, France) were used.

Animals were anesthetized using an intraperitoneal injection of ketamine (75 mg/kg; Imalgene^®^, Merial, Lyon, France) and medetomidine (0.5 mg/kg; Medetor^®^, Virbac, Burgdorf, Germany) and allocated into six groups of 5 animals: one group of non-induced and non-treated animals (naïve control), a group of carrageenan-induced but non treated animals (negative control), a group of animals induced with carrageenan and treated with a gel containing etofenamate-SLN (0.6% etofenamate corresponding to 1.2 mg etofenamate/paw), a group of animals induced with carrageenan and treated with a gel containing ibuprofen-SLN (0.3% ibuprofen corresponding to 0.6 mg ibuprofen/paw), a group of animals induced with carrageenan and treated with an etofenamate commercial gel (Reumon Gel^®^ 5%) corresponding to 10 mg etofenamate/paw, and a group of animals induced with carrageenan and treated with a commercial ibuprofen gel (Ozonol^®^ 5%) corresponding to 10 mg ibuprofen/paw. Figure 1 schematically represents the experimental time course. Gels (0.2 g) were applied topically to the right hind paw of anesthetized animals. Thirty minutes later, 0.1 mL of 1% carrageenan saline solution was injected into the subplantar area of the right hind paw of all animals [20]. The evaluation of paw edema was monitored by changes in both paws by water displacement method using a plethysmometer (Ugo Basile Srl, Gemonio, Italy) immediately before gel application and 5 h after injection of carrageenan. The percentage of edema (% edema) was calculated as follows:

% Edema = (V_5_ − V_0_)/V_0_ × 100, (3)

where V_0_ and V_5_ are the hind paw volumes before treatment and 5h after carrageenan injection, respectively.

After the experiment, animals were sacrificed, and the paws were excised and fixed in 10% neutral buffered formalin (Sigma-Aldrich) for hematoxylin and eosin (H&E) staining. The slides were examined microscopically (Leica, Axioscope camera Software Leica IM50 Image Manage, Wetzlar, Germany). Histopathological appearance of tissues was compared by changes in structure, edema, and infiltration of inflammatory cells (mononuclear cells and/or polymorphonuclear cells) in the inflammation phase of the dermis.

### 2.14. Statistical Analysis

The results were expressed as mean ± standard deviation (mean ± S.D.) from three independent experiments, except otherwise specified. For the in vivo assay, one-way ANOVA and Bonferroni multiple comparisons tests were performed to determine the difference between edema values among the groups (α = 0.05).

## 3. Results and Discussion

### 3.1. Preparation and Characterization of Etofenamate and Ibuprofen-Loaded SLN

**Figure 2:**
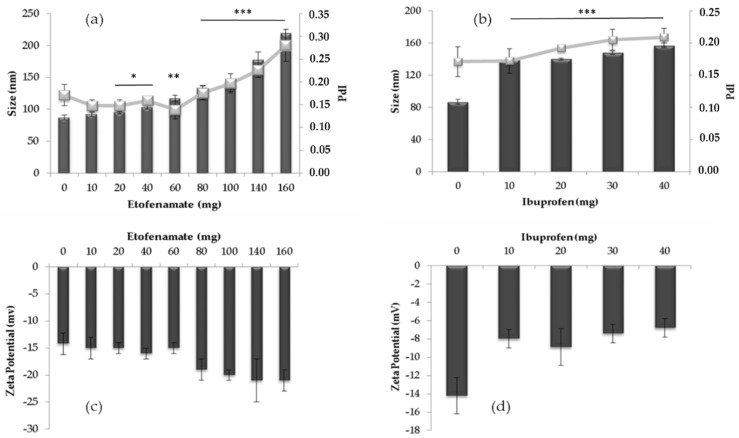
(**a**,**b**) Mean particle size (bars) and polydispersity index (PdI) (lines) and and (**c**,**d**) zeta potential of etofenamate-SLN and ibuprofen-SLN. Statistical significance is presented only for size. All the data were compared to the absence of a drug. The level of significance was set at the probabilities of * *p* < 0.05, ** *p* < 0.01 and *** *p* < 0.001. Results represent the mean ± S.D.; *n* = 3.

Different amounts of the studied drugs were incorporated into SLNs in order to evaluate the effect of the initial drug concentration on the particle size distribution and zeta potential values, as well as on the incorporation parameters (Figure 2).

In general, the increase in the amount of drug led to an increase in the mean particle diameter and PdI (Figure 2a,b). In the case of etofenamate, it was possible to distinguish the existence of two distinct regions: 10–60 mg and 60–160 mg. The increase in the mean particle diameter and the PdI associated with an increase (absolute value) of zeta potential (Figure 2c) observed at the limit of these two regions, which suggests a matrix saturation leading to the progressive accumulation of the drug in the outermost layers of the particle or even on its surface. The final particle size is dependent on several parameters such as the type and concentration of lipids, the type, and concentration of particle stabilizers, and the preparation method employed. Larger particle sizes were obtained for etofenamate-SLNs when increasing the lipid amount [3]. In the same study, Badilli et al. observed a decrease in particle size with the increasing concentrations of Tween^®^ 80 [3]. The fact that etofenamate is a liquid substance at room temperature is consistent with the formation of a Type II SLN structure (drug-enriched shell), where the progressive accumulation of drug molecules at the surface of the particles changed the zeta potential for more negative values (Figure 2c) [21].

In the case of ibuprofen, the inclusion of higher drug amounts caused a decrease in the zeta potential absolute value from −14 ± 4 to −7 ± 3 mV, a value that remained practically constant regardless of the amount of drug added (Figure 2d). Other authors have reported smaller particle sizes for ibuprofen-SLNs obtained by hot-melt extrusion [22] and more comparable values when using the same preparation method [23]. The ibuprofen molecule has a melting range (75–78 °C), slightly higher than that of Compritol^®^ 888 ATO (69–74 °C), which may justify the formation of a Type III SLN structure (lipid-enriched shell), or even the formation of Type I SLN structure (homogeneous matrix), given the proximity of the melting ranges [21]. In both theoretical structures, the amount of drug present in the formulation should not extremely influence the zeta potential.

**Figure 3:**
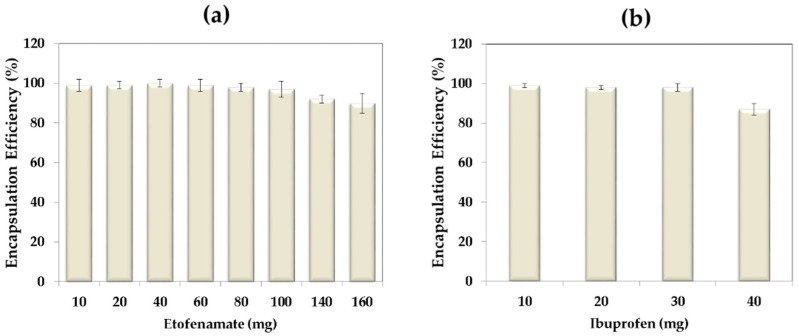
Encapsulation efficiency of etofenamate (**a**) and ibuprofen (**b**) in SLN. Values represent the mean ± S.D.; *n* = 3.

The EE% of etofenamate and ibuprofen in SLN was determined by studying the influence of the initial drug amount. Drugs were incorporated into the particles at different increasing amounts. As presented in Figure 3, EE% was higher than 90% and 97% for etofenamate and ibuprofen, respectively. For etofenamate, these values are in accordance with the literature [6,7], but for ibuprofen, they were similar to those obtained by Badge et al. [22] and almost twofold the values reported by Pham et al. [23].

In the case of etofenamate (Figure 3a), for drug amounts higher than 60 mg, the EE% gradually decreased. In the case of ibuprofen (Figure 3b), the EE decreased for an initial amount higher than 30 mg. Based on the results obtained for size and drug incorporation, 60 mg etofenamate and 30 mg ibuprofen were selected as initial drug amounts to prepare etofenamate- and ibuprofen-loaded SLNs and to conduct the further drug-carrier system characterization. Other authors have achieved comparable EE% for etofenamate [6,7] and smaller EE% for ibuprofen [22,23]. However, The comparison between studies is difficult to establish as a small modification of the tested conditions may assume a great impact on drug encapsulation.

### 3.2. DSC Thermal Analysis

**Figure 4:**
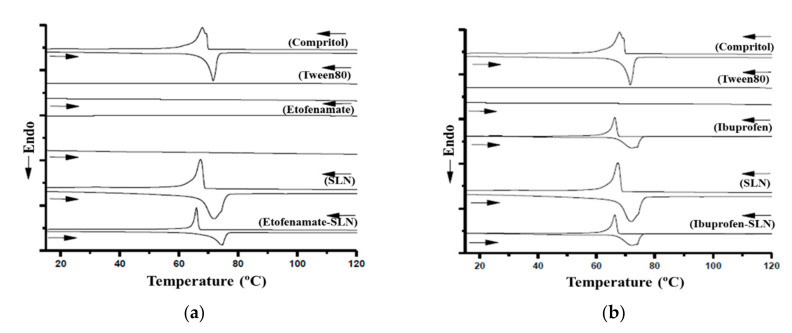
Differential scanning calorimetry (DSC) thermograms of etofenamate-loaded SLNs and individual components (**a**) and of ibuprofen-loaded SLNs and individual components (**b**). Compritol: Compritol^®^ 888 ATO.

Drug-loaded SLN and the individual components of the drug-carrier system were analyzed by DSC. Figure 3 shows the thermograms of free drug, individual particle components, and empty and loaded SLNs for etofenamate (Figure 4a) and ibuprofen (Figure 4b). In both cases, SLNs were prepared with Compritol^®^ 888 ATO. Thermograms of drugs and Tween^®^80 did not show any thermal singularity at the studied temperature range, while the solid lipid thermogram showed a melting event (72 ± 3 °C) that corresponds to that described in the literature (Ph. Eur. [17]). This DSC profile remained unchanged in the thermograms of empty SLNs and SLNs containing etofenamate. Thus, the drug nanoencapsulation did not influence the lipid fusion, which again corroborates the formation of a Type II SLN structure.

A similar analysis was performed for the SLNs formulated with ibuprofen (Figure 4b). After drug incorporation into SLNs, the melting point of the matrix slightly increased from 70 to 72 °C, which is in line with reports that ibuprofen nanoencapsulation in SLN made of glyceryl palmitostearate (Precirol^®^) results in a slight increase in the melting point, due to the interaction between the lipid phase and the drug [24]. This phenomenon had been earlier reported for SLN made of glyceryl stearate (Imwitor^®^ 900) containing a poorly water-soluble drug [25]. The authors explained it as being due to an interaction between the lipid phase and the drug, indicating that the drug was, at least in part, loaded into the particles because the phenomenon was not observed in physical mixtures of lipid-drugs.

### 3.3. DLS Thermal Analysis

DLS thermal analysis is often used to study the physical behavior of SLN suspensions during processes that include heating and cooling variations. In the present work, it was used as a stress test that provided useful information about the physical stability of the formulations. During the heating phase (Figure S1a), for the temperature range between 69–74 °C, which corresponds to the melting range of the lipid, a change in the mean particle diameter was observed from 102 (66 °C) to 88 nm (77 °C). This means the particle diameter value remained stable until the end of the heating phase. During the cooling phase, the mean diameter of the particles remained between 80 nm and 90 nm until 60 °C. Once reaching this temperature, the size increased to its initial value (102 nm at 55 °C), which remained unchanged until the end of the cycle. The PdI value did not vary significantly (*p* < 0.05) throughout the study.

In the case of drug-loaded SLNs, the heating curves (Figure S1b,c) showed a similar profile as that described by empty SLNs. Etofenamate-SLNs had their mean size reduced from 106 to 94 nm at 62 °C, and ibuprofen-SLNs had their mean size reduced from 147 to 131 nm, at the same temperature. However, during the cooling phase, drug-containing SLNs did not recover their initial diameter. Furthermore, at the end of the heating and cooling cycles, both the drug-containing SLN preparations presented a precipitate, which was found to correspond to the incorporated drugs. During the heating phase, a reorganization of the lipid matrix to form particles of smaller dimensions might have occurred, which is indicative of the instability of these formulations at high temperatures [26]. According to the literature, when heating drug-loaded SLN dispersions, the water solubility of drugs increases, and the drugs may diffuse from the molten lipid matrix to the aqueous phase [27]. During the cooling phase, the matrix begins to crystallize while relatively large amounts of the drug remain in the aqueous phase. The continued cooling process leads to drug supersaturation in the aqueous phase, which tends to diffuse again into the lipid phase where a solid core has already been formed, being only the outermost liquid layer left to accommodate the drug. The PdI values also decreased at the end of the heating/cooling cycle (from 0.176 to 0.083 for etofenamate-SLNs and from 0.183 to 0.088 for ibuprofen-SLNs).

### 3.4. Morphological Characterization

**Figure 5:**
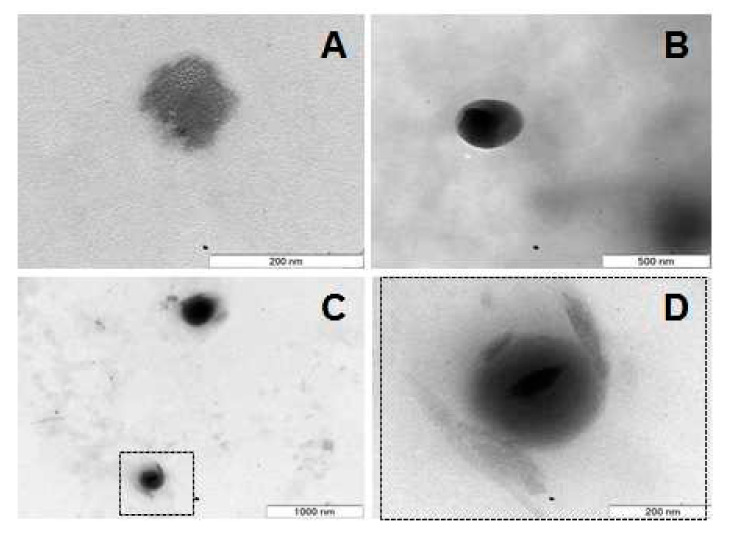
Micrographs obtained by TEM, where solid particulate material is evident: an empty SLN (**A**), an etofenamate-SLN (**B**), and an ibuprofen-SLN (**C**,**D**). Image (**D**) corresponds to a zoom of the dashed outlined rectangle from image (**C**). Scale bars: 200 nm (**A**,**D**), 500 nm (**B**), and 1000 nm (**C**).

Nanoparticle morphology was analyzed by TEM and AFM. TEM analysis showed that empty SLNs had an irregular shape (Figure 5A) and that drug-containing SLNs were spherical (Figure 5B,C). The mean particle diameter was found to be between 80 and 90 nm for empty particles and between 100 and 220 nm for drug-containing nanoparticles.

In the case of ibuprofen-SLN (Figure 5D) it was possible to observe the appearance of crystals on the surface of the particle, which was probably due to the eventual drug precipitation as a consequence of the crystalline reorganization of the lipid matrix. The crystallization tendency and polymorphic transitions in triglyceride nanoparticles between the three crystal structures (i.e., α hexagonal, unstable; β′ orthorhombic, metastable; β triclinic, stable) have been described and visualized by imaging studies [13,27]. After SLN preparation, part of the lipid crystallizes in high energy forms α or β′. During storage, these unstable forms tend to convert into the lowest free energy form β and are therefore thermodynamically more stable. As this form presents a more ordered crystalline structure, with fewer imperfections, it has less capacity to accommodate drug molecules inside. Thus, the polymorphic transitions of the solid lipid matrices generally lead to drug expulsion and SLN instability [13].

**Figure 6:**
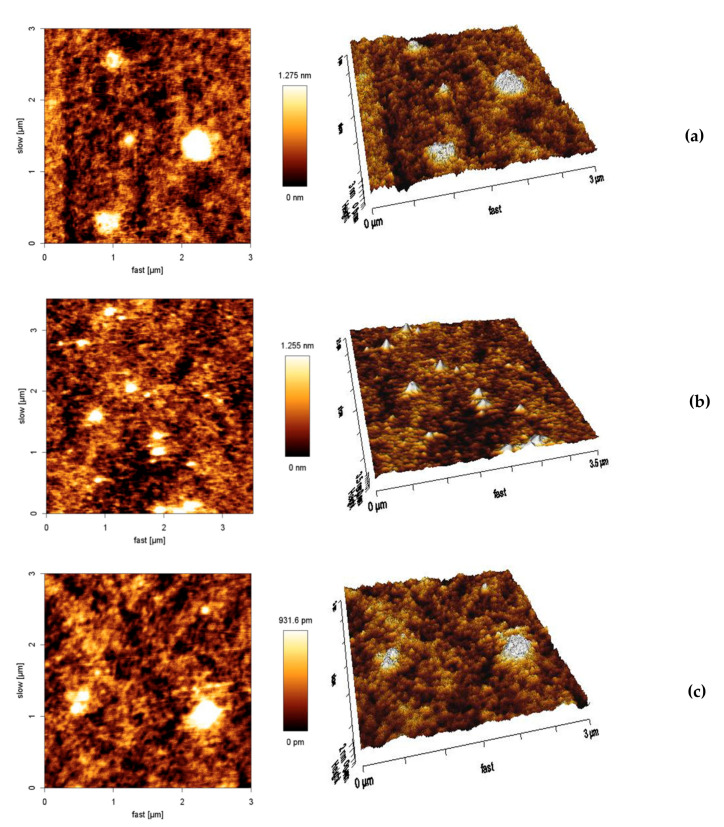
AFM images of empty SLNs (**a**), etofenamate-SLNs (**b**), and ibuprofen-SLNs (**c**).

AFM was also used to provide information about the morphology, diameter, and particle size distribution of the developed nanosystems. Figure 6 shows the cross-sections and the 3D images of empty SLNs, etofenamate-SLNs, and ibuprofen-SLNs.

The mean particle diameters obtained by AFM were similar to those established from the DLS and TEM analyses. A thorough inspection of cross-section profiles and 3D imaging indicates that the particles have an irregular shape and rough surface consistent with the images obtained by TEM for empty nanoparticles (Figure 6A). No differences were observed between empty and loaded particles. In fact, the combination of several analytical methods gave complementary information for the characterization of SLNs. Using different techniques, it was found that drug encapsulation had no major impact on particle dimensions or morphology.

### 3.5. In Vitro Cytotoxicity and Internalization Studies

**Figure 7:**
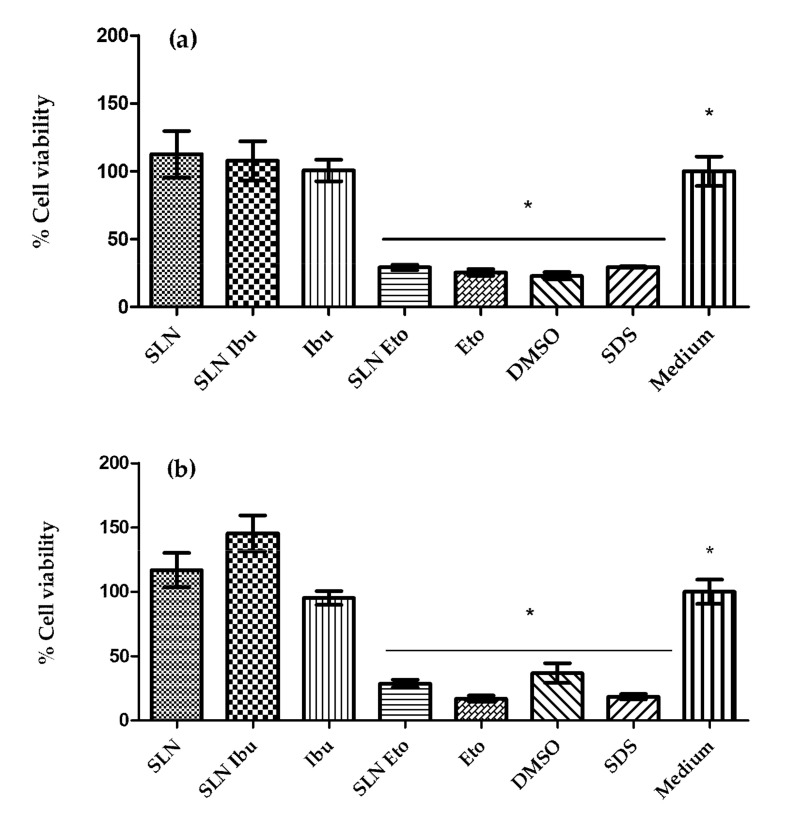
Cell viability ((**a**)-Df and (**b**)-HaCaT cell lines) after 24 h of incubation with 10 µL of empty SLNs (SLN), ibuprofen loaded-SLNs (SLN Ibu) (3 mg/mL), etofenamate loaded-SLNs (SLN Eto) (6 mg/mL), etofenamate solubilized in DMSO (Eto) (1.2 mg/mL), ibuprofen solubilized in DMSO (Ibu) (3 mg/mL), DMSO (dimethyl sulfoxide), SDS (sodium lauryl sulfate) (10 mg/mL), and cell culture medium (Medium). Results represent the mean ± S.D., *n* = 9; * *p* < 0.05 vs medium.

Cell viability was assessed using two human cells from skin fibroblasts (Df cell line) and keratinocytes (HaCaT cell line) in an MTT assay. Empty SLNs at the concentration tested did not reduce the viability of either cell line (Df and HaCaT), showing values of 112 ± 17% and 117 ± 13% for the Df (Figure 7a) and the HaCaT cell lines (Figure 7b), respectively. Regarding the ibuprofen-loaded SLNs (SLN Ibu), this value did not change significantly (*p* > 0.05). Therefore, the drug carrier association did not reduce the viability of either cell line (Df and HaCaT), showing values of 107 ± 14% and 145 ± 14% for the Df (Figure 7a) and HaCaT cell lines (Figure 7b), respectively. In turn, free and encapsulated etofenamate reduces viability in either cell line (Df and HaCaT), showing values of 29 ± 2% and 28 ± 1% for the Df and HaCaT cell lines, respectively. According to the Environmental Health and Safety Publications Series on Testing and Assessment (OECD TG 439), a substance is defined as an irritant if cell viability is less than 50% for an exposure time between 15–60 min in an in vitro test system of reconstructed human epidermis, which closely mimics the biochemical and physiological properties of the upper parts of the human skin, i.e., the epidermis [28]. In the present study, cells were exposed to samples for 24 h, with cell viability values greater than 50%, both for empty SLNs and for ibuprofen-SLNs. Thus, according to these criteria, this formulation may be considered non-irritating. The etofenamate-SLNs can be considered irritating according to the same standard, although this test must be repeated in cells mimicking the skin organization of the skin to allow drawing sounder conclusions. However, in terms of safety, these findings are in line with previous publications, suggesting a low toxicological risk for the SLN formulations [29].

**Figure 8:**
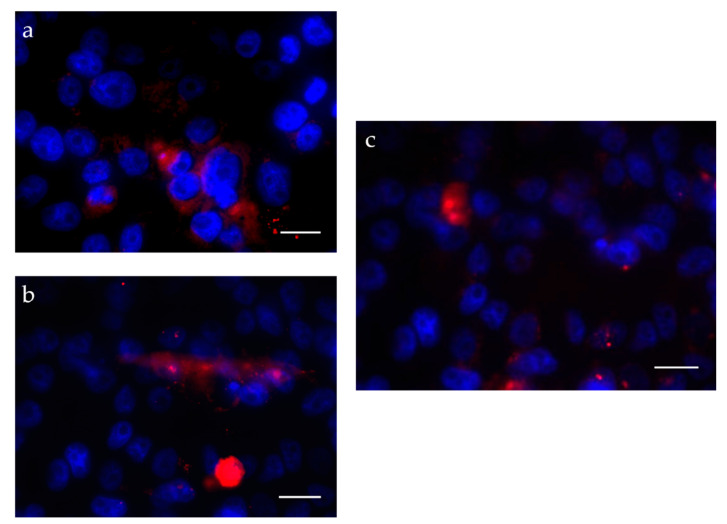
Fluorescent micrographs of SLN in HaCaT cells: (**a**) empty SLN, (**b**) etofenamate-loaded SLN, (**c**) ibuprofen-loaded SLN. (Scale bar 50 µm).

The internalization of fluorochrome-labeled SLNs (Nile red) was studied in HaCaT cells and evaluated by fluorescence microscopy (Figure 8).

After 1 h of incubation, the drug-loaded SLN could be observed inside the cells, mainly in the cytoplasm. The same results were observed by Küchler et al., showing that after 0.5 h almost 50% of the 150–170 nm SLNs, prepared with Compritol^®^ 888 ATO, labeled with the same fluorochrome (Nile red) were found inside the human keratinocytes [30].

### 3.6. Drug-Loaded Hydrogel Physicochemical Characterization, Microbiological Efficacy, and Stability

Drug-loaded SLN suspensions were gelated instead of adding a preformed hydrogel, using an up-scalable process. Gels were prepared with HPMC as a thickener, propylene glycol as a humectant and drug solubilizer, ethyl alcohol as a preservative, and menthol as a skin permeation enhancer [16]. Propylene glycol activity as co-solvent in topical lipophilic ibuprofen formulations has been studied, and it has been observed that the penetration of the skin cells by propylene glycol is proportional to its concentration, affecting the solubility of ibuprofen in the skin barrier [31].

The physical stability of lipid nanoparticle suspensions is greatly influenced by the type of lipids used, as well as the type of emulsifier present in the formulation [32]. These parameters were also considered to play a critical role in the stability of SLNs intended for further processing, such as sterilization or freeze-drying [32]. Placebo formulation (hydrogel) was transparent while the SLN-containing hydrogels (empty and drug-loaded) remained opaque, homogeneous pearl-white throughout the 12 months. Drug recovery from formulation throughout the study was almost complete for both drugs, even under intermediate and accelerated conditions (Table 2), thus confirming the physical robustness of the SLN formulations observed during stress testing by DLS. The variability obtained for drug recovery, probably due to the batch size, was within the specifications (100 ± 5%). All the formulations showed shear-thinning behavior meaning that viscosity decreased as a function of shear rate applied. This behavior is ideal for topical application because when applying a force, the viscosity decreases, increasing the spreadability of the formulation. Identical results were reported for a hydroalcoholic gel containing 5% (*w/w*) etofenamate for topical administration [33]. The inclusion of ethanol seemed to decrease the resistance to structural breakdown.

The pH values suitable for skin applications range between 4.5 and 5.5. Just after preparation, the formulations presented a mean pH value of 5.51 ± 0.01 for empty SLN hydrogel, 5.6 ± 0.1 for the etofenamate-SLN hydrogel, and 4.4 ± 0.1 for the ibuprofen-SLN hydrogel.

After gel preparation, there was a change in SLN particle diameter, from 81.20 µm ± 0.08 to 6.91 µm ± 0.24 (after 12 months), which can be attributed to an interaction between gel components and the particles (data not shown), promoting aggregation. This raises the hypothesis of a structural reorganization of the SLN-hydrogel system during this period, which results from competition for water between the HPMC chains and the surface of the surfactant-stabilized SLN (Tween 80^®^). Polymer chains have greater water molecule retention capacity than the surface of the nanoparticles, and there will be less water available to surround SLNs. Thus, the structure will evolve from a homogeneous SLN-hydrogel system with the trapped nanoparticles forming “aggregate domains” with a relatively uniform distribution, suggesting that this interaction may result in an increase in SLN particle diameter and a decrease in gel viscosity [23]. In general, there was a decrease in viscosity observed after 12 months for both etofenamate-SLN hydrogel and ibuprofen-SLN hydrogel at all tested temperature conditions (Table 2). A decrease in apparent viscosity, more pronounced as temperature increases, was previously observed, in agreement with the depolymerization of cellulose polymers with prolonged heating [34]. Gels containing drug-loaded particles exhibited behavior like empty SLN hydrogels (data not shown), suggesting that drug incorporation did not affect the physical stability of the system.

The effect of the hydrogels on the viability of two human cell lines derived from skin, i.e., fibroblasts (Df cell line) and keratinocytes (HaCaT cell line), was assessed as described in Section 2.8. The hydrogel formulation containing empty SLN did not affect the viability in any of the cell lines showing values of 97 ± 8% and 111 ± 6% for the Df (Figure S2a) and HaCaT cell line (Figure S2b), respectively. The effect of hydrogel containing ibuprofen-loaded SLN (Gel SLN Ibu) was not significantly different from that of empty particles (*p* > 0.05). Therefore, ibuprofen-loaded SLN did not reduce the cell viability of both cell lines (Df and HaCaT), presenting a value of 139 ± 17% and 91 ± 10% for Df (Figure S2a) and HaCaT cell line (Figure S2b), respectively. The hydrogel containing etofenamate-loaded SLNs showed cytotoxicity (cell viability < 50%) for both cell lines, which was comparable to the effect of the same amount of drug in the etofenamate commercial gel. Introducing SLN in the formulation had no impact on cell viability, presenting similar viability levels for the two cell lines tested. Therefore, the formulations can be considered to be non-irritating, according to the OECD [28].

Microbiological testing revealed that etofenamate-SLN hydrogel and ibuprofen-SLN hydrogel were stable for 12 months as the total aerobic microbe, yeast, and mold counts were within accepted values following the established criteria (<10 cfu/g). In addition, there was no observable increase in the number of viable micro-organisms between days 14 and 28; thus, the recommended efficacy was achieved in both formulations. The efficacy of antimicrobial preservatives was not compromised, providing adequate protection against microbial contamination.

### 3.7. In Vitro Studies of Percutaneous Absorption

**Figure 9:**
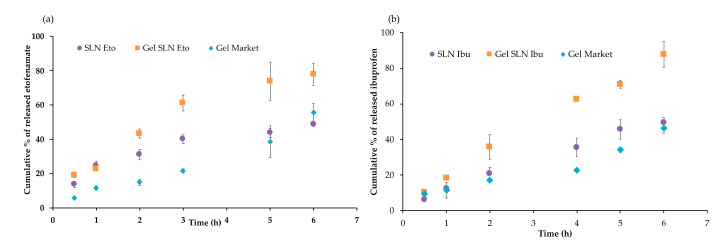
Release profiles of etofenamate (**a**) and ibuprofen (**b**) incorporated in SLN and SLN-gels, and commercial formulations (Gel Market) corresponding to Reumon^®^ Gel 5% (etofenamate) and Ozonol^®^ 5% (ibuprofen), through a Tuffryn^®^ membrane, after 6 h. Results are expressed as mean ± S.D.; *n* = 6.

The release profiles of etofenamate and ibuprofen incorporated in SLNs and SLN-hydrogels are shown in Figure 9.

Concerning etofenamate release, 43 ± 3% of the drug in the formulation was released from the SLN after 6 h, corresponding to 534 ± 6 μg cm^−2^. As can be observed, the drug release was almost constant along time, although faster in the first 2 h, a result also confirmed by the zero-order mathematical model, which better describes this release profile (R^2^ = 0.992). The faster initial release (37 ± 3.8% after 1 h) was consistent with the accumulation of crystals at the surface of the nanoparticles, observed by TEM (Figure 5D). The incomplete drug release from the SLNs at 6 h may be due to a chemical affinity of etofenamate to the lipid matrix of the SLNs that can cause the partial retention of the drug by the vehicle, decreasing the amount available for diffusion (Figure 9a). This is in agreement with the calorimetric studies of the interaction between SLNs and the model drug indomethacin, reported by Castelli et al. [35].

The differences between the release profiles of etofenamate and ibuprofen from SLNs may be due to their different solubility in water but also related to the different SLN structures, as previously discussed. Both drugs are virtually insoluble in water; however, etofenamate solubility (0.0012 mg mL^−1^) is even lower than that of ibuprofen (0.041 mg mL^−1^) [36]. Also, the differences between partition coefficients (LogP = 4.99 for etofenamate and 3.72 for ibuprofen) [37] may have a critical effect on the release rate [38]. Nevertheless, although some drug partitioning out of SLN cannot be disregarded during gel formation, release profiles suggest the drugs are mostly associated to the nanoparticles, particularly the highly lipophilic liquid drug etofenamate.

The release profile of ibuprofen follows the Higuchi model, with a higher diffusion in the first hours, followed by a constant diffusion (R^2^ = 0.989). Release kinetics were in agreement with the majority of the data reported in the literature regarding the release profiles of drugs encapsulated in nanoparticles when using Franz diffusion cells [39,40].

The release of ibuprofen from gels after 6 h was 87.91 ± 7.22% and 46.39 ± 2.97% for the SLN hydrogel and the commercial gel, respectively. Data analysis revealed a zero-order kinetic model, with significant differences (*p* < 0.05) between the two gels, after 2 h. As observed for etofenamate, this difference may be due to the different viscosities and/or to the excipients of the formulations. Additionally, it is possible that some crystallization of the ibuprofen in the reference gel might have difficulty in diffusing across the membranes [41,42].

Drug release from SLN incorporated in hydrogels showed that, after 6 h, the release of etofenamate was 77.86 ± 6.52%, compared to 55.39 ± 5.20% for the commercial gel. Both gels presented zero-order release kinetics but different profiles due to the different characteristics of both gels. In fact, the SLN-containing hydrogel had a lower viscosity compared to the commercial gel, which facilitates the migration of the drug throughout the formulation [43,44]. Furthermore, gel excipients may influence the release profiles: propylene glycol and ethanol, which have a great affinity for the receptor phase (ethanol:water 40:60 *v/v*) and might have increased the diffusion from this system [45,46].

**Figure 10:**
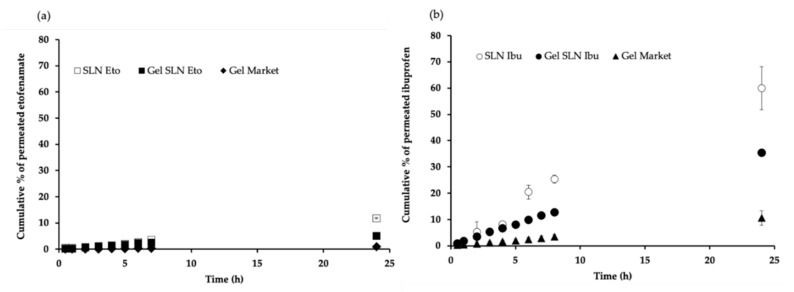
Permeation profiles of (**a**) etofenamate-SLN (SLN Eto), etofenamate-SLN hydrogel (Gel SLN Eto), and etofenamate reference gel (Gel Market-Reumon^®^ Gel 5%) and (**b**) ibuprofen-SLN (SLN Ibu), ibuprofen-SLN hydrogel (Gel SLN Ibu), and ibuprofen reference gel (Gel Market-Ozonol^®^ 5%) through human skin cells (SC). Results are expressed as mean ± S.D.; *n* = 6.

Figure 10 shows the permeation profiles of etofenamate and ibuprofen from the SLNs, SLN hydrogel, and from the reference gel through human SC for 24 h.

Similar to what was suggested by the release profiles obtained with the synthetic membrane, drug permeation through the SC revealed differences between the drugs nanoencapsulated in SLNs. While etofenamate-SLNs showed a drug permeation of only 2.00 ± 0.22% after 24 h (Figure 10a), ibuprofen-SLN showed a permeation of 14.00 ± 0.24% (Figure 10b) in the same period. Larger, more lipophilic solutes display slower diffusivity values [47]. The relationship between solute lipophilicity and skin permeation shows that vehicle:stratum corneum partition coefficients are related to logP, and the concentration of solutes in each of the tissue layers appeared to increase with lipophilicity [47], which is in line with our findings and is supported by our data.

The SC is a much more selective membrane, and it is much more difficult to overcome. The vehicle in which the active substance is incorporated has an enormous influence on the penetration and permeation across the cutaneous tissue. The direct analysis of the profiles obtained shows that the permeation capacity of both drugs is low, and these results were similar to those obtained for other lipophilic molecules encapsulated in Compritol^®^ 888 ATO nanoparticles [48]. This might be directly related to the different characteristics of the lipid dispersions used. However, percutaneous absorption does not exclusively depend on the physicochemical properties of the vehiculated substance, being also highly influenced by the vehicle properties, as shown elsewhere [42,49].

The permeability rate constant Kp, the flux J, and the lag time (TL) were calculated from the linear portion of the permeation curves of the different formulations (Table 3).

Permeation data show significant differences between the SLN hydrogel and the reference gel for etofenamate. The Kp was significantly higher for the etofenamate-SLNs gel (5.12 × 10^−3^ cm h^−1^) compared to the commercial reference gel (2.35 × 10^−3^ cm h^−1^), while the flux was higher for the reference gel (11.97 µg cm^−2^ h^−1^) compared to the etofenamate-SLN hydrogel (6.15 µg cm^−2^ h^−1^). In the case of ibuprofen, there were also significant differences, but in this case, both the Kp and the flux were higher for the reference gel (2.98 × 10^−2^ cm h^−1^ and 22.85 µg cm^−2^ h^−1^, respectively) compared to the same parameters for the ibuprofen-SLN hydrogel (1.16x10^−2^ cm h^−1^ and 17.55 µg cm^−2^ h^−1^, respectively). The higher fluxes observed for the reference gels are possibly due to the higher drug concentration in these gels. It also explains the higher cutaneous permeation and the higher lag time observed in both cases.

Both drug-containing SLN hydrogels showed a higher permeation capacity compared to the reference drug products. Thus, the explanation of drug release profiles can also be applied to the permeation studies. The presence of ethanol increased in 10% ibuprofen permeation across a silicone membrane and across the SC [50]. Morimoto et al. studied the influence of ethanol and menthol in the permeation of flurbiprofen in rabbits and observed a high absorption ratio when compared with gels without these permeation enhancers [51]. The addition of terpenes to the lipid nanoparticle formulations containing ethanol increased the permeation compared to just ethanol which is, by itself, a permeation enhancer [16,52].

### 3.8. In Vivo Evaluation of the Anti-Inflammatory Effect of Etofenamate-SLN Sel and Ibuprofen-SLN Gel

**Figure 11:**
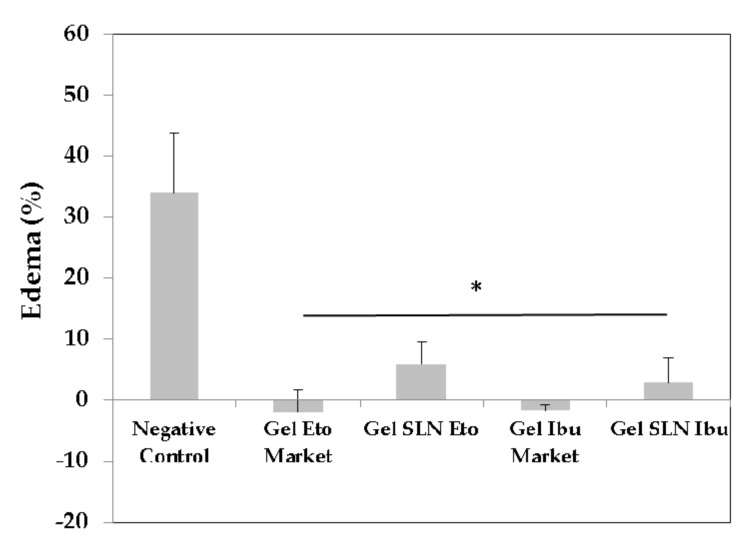
Paw edema (%), 5 h after carrageenan injection in different test groups: induced non treated animals (Negative control), etofenamate reference gel (Gel Eto Market-Reumon^®^ Gel 5%), etofenamate-SLN hydrogel (Gel SLN Eto), ibuprofen reference gel (Gel Ibu Market-Ozonol^®^ 5%) and ibuprofen-SLN hydrogel (Gel SLN Ibu). Data represent the mean ± S.E.M.; *n* = 5. * *p* < 0.01 vs. negative control.

The anti-inflammatory effects of etofenamate-SLN hydrogel and ibuprofen-SLN hydrogel were evaluated in the carrageenan-induced paw edema rat model (Figure 1) using, as references, the commercial topical gels of etofenamate and ibuprofen. Carrageenan injection produced a paw volume increase of 34% (negative control), which was significantly mitigated by the topical application of the different gels containing the anti-inflammatory drugs tested (Figure 11).

The formulations tested showed significantly inhibited (*p* < 0.05) carrageenan-induced paw edema (Figure 11). All treated groups were significantly different from the Negative control group. However, drug-loaded gels were not different from the respective commercial reference and were not different from each other. The treated animals presented −2.0 ± 3.7% and 5.8 ± 3.7% edema for the etofenamate commercial reference gel and etofenamate-SLN gel, respectively. Etofenamate reference gel has higher drug content (5%) than etofenamate-SLN gel (0.6%). The amount of gel applied to animal paws was kept constant (0.2 g) between the different groups. This means that animals treated with reference gel received 10 mg etofenamate on the paw, and animals treated with etofenamate-SLN gel received only 1.2 mg etofenamate. This result was very encouraging as the same anti-inflammatory effect was obtained with a dose 8.8 times lower when using a controlled release formulation. Previous publications only report data from in vitro anti-inflammatory activity of etofenamate [53]. Regarding ibuprofen gels, there was also a marked attenuation of the edema, either in animals treated with ibuprofen commercial reference gel or ibuprofen-SLN gel (Figure 11). The results clearly show that the ibuprofen formulations tested inhibited (*p* < 0.05) the hind paw edema in comparison with the Negative control. Treated animals presented edema percentage values of 1.7 ± 0.9% and 2.9 ± 4% for ibuprofen reference gel and ibuprofen-SLN gel, respectively. As observed for etofenamate gels, the ibuprofen reference gel contained a higher drug concentration than the ibuprofen-SLN gel that resulted in 0.6 mg ibuprofen being applied to the paw of animals treated with a dose of ibuprofen-SLN gel and 10 mg ibuprofen in the group treated with ibuprofen reference gel. The same anti-inflammatory activity was achieved with a dose 16.6 times lower when treating animals with ibuprofen-SLN hydrogel. The nanoencapsulation of these anti-inflammatory drugs in SLN resulted thus in a promising approach to reduce the drug concentration in the final preparation. The higher anti-inflammatory effect achieved for ibuprofen might be due to the high drug accumulation at the inflammation site, as a result of a higher permeation achieved for SLN based formulations (Figure 10). The superiority of the anti-inflammatory effect of ibuprofen once encapsulated in SLN in contrast to the conventional gel has been previously reported [22]. A recent study also demonstrated the superiority of local anti-inflammatory and analgesic activities of ibuprofen-SLN gels in a rat model of rheumatoid arthritis when using the same dose (5%) of commercial gel tested for comparison [23].

To the best of our knowledge, we report here for the first time in vivo data regarding the anti-inflammatory activity of etofenamate-SLN hydrogels in a carrageenan edema model. Badilli et al. showed higher inhibitory activity of COX for semisolid etofenamate-SLNs in comparison with pure etofenamate; however, no in vivo data has corroborated such promising findings [53,54].

**Figure 12:**
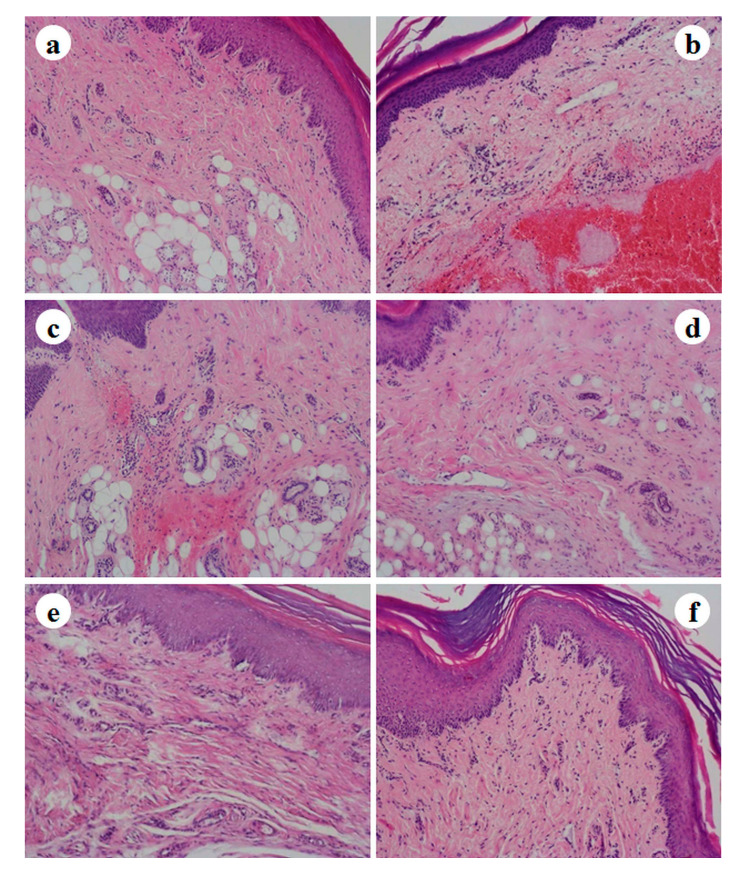
Representative photomicrographs from rat paw histological sections stained with H&E of (**a**) naïve control, (**b**) negative control, (**c**) etofenamate reference gel (Reumon^®^ Gel 5%), (**d**) etofenamate-SLN hydrogel, (**e**) ibuprofen reference gel (Ozonol^®^ 5%), and (**f**) ibuprofen-SLN hydrogel. Magnification: 100×.

Histological analysis of the rat paws subjected to carrageenan injection and topical treatment with anti-inflammatory drugs was performed after edema experiments. Carrageenan injection produced changes in the epidermal layer, intense edema characterized by an increased thickness of the epidermis, a hemorrhage in the periphery of the dermis in which small foci of cellular infiltration was observed, especially neutrophils and dispersed mast cells (Figure 12b). Similar results were observed by other authors and described in previously published studies [55]. Figure 12d corresponds to a paw of an animal induced with carrageenan and treated with etofenamate-SLN gel. In this case, there was an absence of changes in the epidDermis and a significant decrease in edema and infiltration of inflammatory cells. Slight connective tissue edema was observed in the dermis. The use of this hydrogel contributed to the prevention of inflammation since gel application occurred prior to carrageenan induction of edema. In this acute model, the test substance was applied half an hour before carrageenan injection so that the drug can be absorbed and is available in the paw tissues at the time inflammation is induced. Using this same animal model, it was possible to observe by histological examination of rat paws lower edema, and fewer inflammatory infiltrates when using a natural extract encapsulated in polymeric nanoparticles in comparison with non-encapsulated extract [56]. The group treated with ibuprofen SLN gel (Figure 12f) presents an absence of changes in the epidermis and a decrease in edema and infiltration of inflammatory cells. The topical application of anti-inflammatory drugs in the different gels resulted in the amelioration of the inflammatory state.

## 4. Conclusions

The work essentially addressed the pharmaceutical and preclinical development of improved topical NSAID-containing formulations consisting of etofenamate and ibuprofen encapsulated in SLNs. Nanoparticle suspensions were then gelated to produce hydrogels suitable for dermal application. All preparation procedures were up scalable and resulted in physically and microbiologically stable formulations. The solid lipid and emulsifying agent, Compritol^®^ 888 ATO and Tween^®^ 80, respectively, resulted in SLN formulations with suitable particle size (<250 nm), encapsulation efficiency (>90%), and stability (12 months). The SLN-containing hydrogels increased drug permeation when comparing etofenamate and ibuprofen hydrogel commercial hydrogel formulations (Reumon^®^ Gel 5% and Ozonol^®^ 5%, respectively). In vivo studies using the rat paw edema inflammation model showed that hydrogels containing lower doses of etofenamate (8.3 times lower) and ibuprofen (16.6 times lower) formulated in SLN produced a similar anti-inflammatory effect, compared to the commercial reference products, with no histological changes in the epidermis and a significant decrease in edema and inflammatory cell infiltration. These studies demonstrate that encapsulation in SLNs associated to a suitable hydrogel is a promising, straightforward technological approach to NSAIDs dermal application. Further optimization studies on SLN physicochemical characteristics, hydrogel rheological behavior, and scale-up are currently being performed.

## Figures and Tables

**Table 1:** Solid lipid nanoparticles (SLNs) hydroalcoholic gel compositions. Eto: etofenamate; Ibu: ibuprofen; Gel SLN: empty SLN hydrogel; Gel SLN Eto: etofenamate-loaded SLN hydrogel; Gel SLN Ibu: ibuprofen-loaded SLN hydrogel.

Formulation	Compritol^®^ 888 ATO (%)	Tween^®^ 80 (%)	HPMC (%)	Propylene glycol (%)	Ethyl alcohol (%)	Menthol (%)	Eto (%)	Ibu (%)	Water (%)
Gel SLN	2.0	0.6	2.0	10.0	10.0	0.1			qs 100
Gel SLN Eto	2.0	0.6	2.0	10.0	10.0	0.1	0.6		qs 100
Gel SLN Ibu	2.0	0.6	2.0	10.0	10.0	0.1		0.3	qs 100

qs: quantum satis.

**Table 2:** Physicochemical stability of drug-loaded hydrogel batches: etofenamate-SLN hydrogel (Gel SLN Eto) and ibuprofen-SLN hydrogel (Gel SLN Ibu) stored at different temperatures (mean ± S.D., *n* = 3).

Time (months)	Gel SLN Eto			Gel SLN Ibu		
	Recovery of Etofenamate (%)	pH	Viscosity (Pa.s)	Recovery of Ibuprofen (%)	pH	Viscosity (Pa.s)
**Batches stored at room temperature (25 ± 2 °C/60 ± 5% RH)**						
0	98.97 ± 0.65	5.60 ± 0.11	41,133 ± 1.80	100.27 ± 0.85	5.64 ± 0.05	46,133 ± 2.30
1	100.89 ± 2.06	5.59 ± 0.12	46,866 ± 3.20	98.31 ± 0.52	5.59 ± 0.03	46,000 ± 7.20
3	99.70 ± 1.63	5.59 ± 0.15	43,133 ± 5.70	102.39 ± 0.37	5.64 ± 0.03	45,933 ± 12.5
6	103.73 ± 1.00	5.42 ± 0.11	42,400 ± 6.50	98.00 ± 4.12	5.67 ± 0.06	38,600 ± 3.70
12	100.50 ± 2.72	5.12 ± 0.16	26,966 ± 2.60	95.09 ± 1.37	5.61 ± 0.04	30,600 ± 24.2
**Batches stored at intermediate conditions (30 ± 2 °C/60 ± 5% RH)**						
0	98.97 ± 0.65	5.60 ± 0.11	41,133 ± 1.80	100.27 ± 0.85	5.64 ± 0.05	46,133 ± 2.30
1	101.38 ± 0.84	5.41 ± 0.07	46,133 ± 4.30	97.36 ± 0.81	4.41 ± 0.07	45,133 ± 3.90
3	100.22 ± 2.80	5.46 ± 0.17	40,466 ± 8.30	102.11 ± 2.39	4.34 ± 0.04	41,233 ± 4.06
6	104.17 ± 0.57	5.50 ± 0.02	43,466 ± 7.70	89.11 ± 4.48	4.40 ± 0.02	34,466 ± 2.89
12	--	--	--	--	--	--
**Batches stored in at accelerated conditions (40 ± 2 °C/75 ± 5% RH)**						
0	98.97 ± 0.65	5.60 ± 0.11	41,133 ± 1.80	100.27 ± 0.85	5.64 ± 0.05	46,133 ± 2.30
1	98.99 ± 0.02	5.43 ± 0.07	38,600 ± 4.40	96.07 ± 2.66	4.43 ± 0.07	47,200 ± 4.20
3	100.50 ± 2.95	5.46 ± 0.14	38,033 ± 2.40	102.07 ± 3.74	4.04 ± 0.57	37,866 ± 8.20
6	99.38 ± 3.83	5.42 ± 0.01	38,733 ± 1.10	91.81 ± 5.56	4.42 ± 0.01	34,200 ± 2.00
12	--	--	--	--	--	--

**Table 3:** Values of permeability rate constant (Kp), flux (J), and lag time (T_L_) obtained for the permeation of etofenamate and ibuprofen across human skin.

Formulation	Kp (cm h^−1^)	J (µg cm^−2^h^−1^)	T_L_ (h)
Etofenamate-SLNs	3.12 × 10^−3^	3.74	3.05
Etofenamate-SLN hydrogel	5.12 × 10^−3^	6.15	1.23
Etofenamate reference gel	2.35 × 10^−3^	11.97	0.65
Ibuprofen-SLNs	5.05 × 10^−3^	4.83	4.04
Ibuprofen-SLN hydrogel	1.16 × 10^−2^	17.55	0.20
Ibuprofen reference gel	2.98 × 10^−2^	22.85	0.36

## Data Availability

Not applicable.

## Supplementary Materials

The following are available online at https://www.mdpi.com/1999-4923/13/3/328/s1, Figure S1: Influence of the temperature on the mean diameter and PdI of studied SLN, as determined by DLS analysis, Figure S2: Cell viability of drug-loaded SLN hydrogels.
