# Supplementary material for: Increased Therapeutic Efficacy of SLN Containing Etofenamate and Ibuprofen in Topical Treatment of Inflammation
Source: Pharmaceutics. 2021 Mar 3;13(3):328. doi: 10.3390/pharmaceutics13030328 (PMC7999628; doi:10.3390/pharmaceutics13030328)
Supplement: Supplementary file 1 [file pharmaceutics-13-00328-s001.pdf]

pharmaceutics

MDPI

Supplementary Materials: Increased Therapeutic Efficacy of SLN Containing Etofenamate and Ibuprofen in Topical Treatment of Inflammation

Giuliana Mancini, Lídia M.D. Gonçalves, Joana Marto, Filomena A. Carvalho, Sandra Simões *, Helena Margarida Ribeiro * and António J. Almeida,*

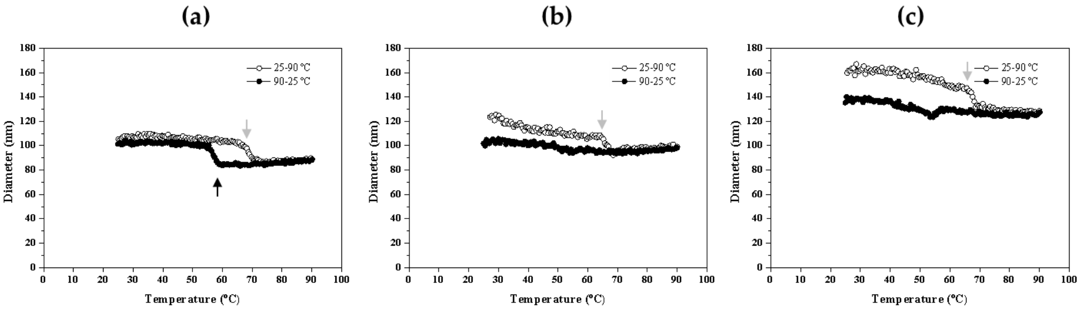

Figure S1. Influence of the temperature on the mean diameter and PdI of empty SLN (**a**), etofenamate-SLN (**b**) and ibuprofen-SLN (**c**), as determined by DLS analysis. (**a**) Empty particles reduce de size at 66 °C (grey arrow) and recover the initial size during the cooling phase at 55 ºC (black arrow); (**b**) Etofenamate-SLN and (**c**) Ibuprofen-SLN reduced the size at 62 °C (grey arrows).

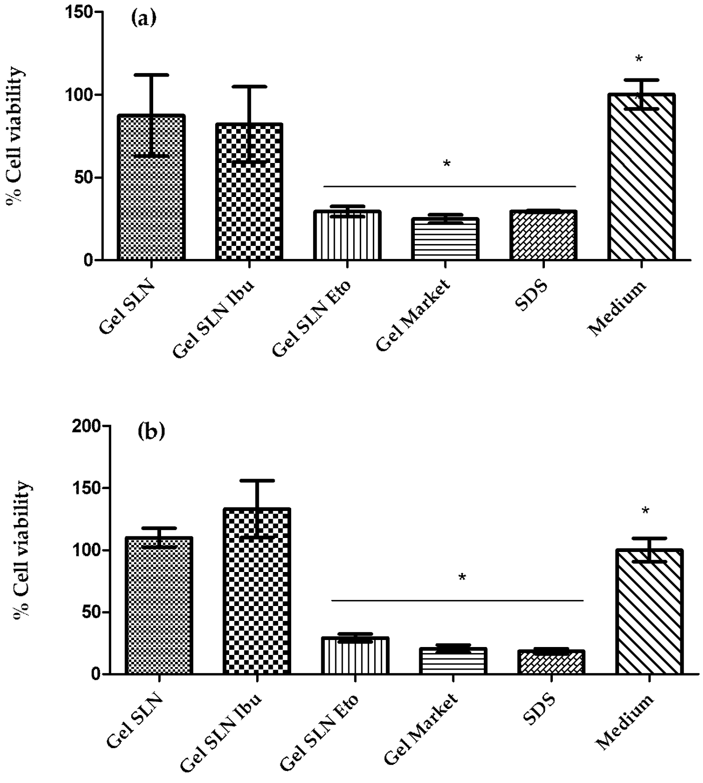

Publisher's Note: MDPI stays neutral with regard to jurisdictional claims in published maps and institutional affiliations.

Copyright: © 2021 by the authors. Submitted for possible open access publication under the terms and conditions of the Creative Commons Attribution (CC BY) license (http://creativecommons.org/licenses/by/4.0/).

Figure S2. Cell viability ((**a**)- Df and (**b**)-HaCaT cell lines) after 24 h of incubation with 10 µL of empty SLN hydrogel (Gel SLN), ibuprofen loaded-SLN hydrogel (Gel SLN Ibu) (3 mg/mL), etofenamate loaded-SLN hydrogel (Gel SLN Eto) (6 mg/mL), commercial etofenamate gel (Gel Market) (6 mg/mL), SDS (sodium lauryl sulfate) (10 mg/mL), and cell culture medium (Medium). Results represent the mean ± S.D., $n = 9$; ** $p < 0.05$ vs medium.
